# Bushenhuoxue Formula Facilitates Articular Cartilage Repair and Attenuates Matrix Degradation by Activation of TGF-*β* Signaling Pathway

**DOI:** 10.1155/2018/2734581

**Published:** 2018-10-08

**Authors:** Rui Dong, Jun Ying, Taotao Xu, Songfeng Hu, Peng Zhang, Chenjie Xia, Liang Fang, Hongting Jin, Pinger Wang

**Affiliations:** ^1^The First Clinical Medical College of Zhejiang Chinese Medical University, Hangzhou 310053, Zhejiang, China; ^2^Institute of Orthopaedic and Traumatology of Zhejiang Province, Hangzhou 310053, Zhejiang, China; ^3^Department of Orthopaedic Surgery, The First Affiliated Hospital of Zhejiang Chinese Medical University, Hangzhou 310006, Zhejiang, China; ^4^Department of Orthopaedics, Shaoxing Hospital of Traditional Chinese Medicine, Shaoxing 312000, Zhejiang, China

## Abstract

**Objective:**

To investigate the effect and underlying mechanism of Bushenhuoxue (BSHX) formula on articular cartilage repair.

**Methods:**

Twenty-four full-thickness cartilage defect rats were divided into two groups: model group and BSHX group (treated with BSHX formula). Macroscopic observation and histopathological study were conducted after 4- and 8-week treatment. Additionally, we also evaluated chondrocyte proliferation, extracellular matrix (ECM) deposition, cartilage degradation, and chondrocyte hypertrophy-related genes expression in chondrogenic ATDC5 cells cultured in BSHX formula-mediated serum. Moreover, we assessed aforementioned genes expression and pSMAD2/3 protein level in Tgf*β*r2 siRNA transfected chondrogenic ATDC5 cells in order to address whether BSHX formula exerts cartilage repairing effect through TGF-*β* signaling.

**Results:**

Neocartilage regeneration promotion effect was observed in cartilage defect rats after BSHX formula treatment, with increases in Col2 and pSMAD2 and decreases in Mmp13 and Runx2. Moreover, cell proliferation, the elevated Col2a1, Aggrecan and pSMAD2/3, reduced Mmp13, Adamts5, Col10a1, and Runx2 expression were also observed in chondrogenic ATDC5 cells cultured in BSHX formula-mediated serum. Besides, the expression alteration of ECM deposition, cartilage degradation, chondrocyte hypertrophy-related genes, and pSMAD2/3 protein levels presented in Tgf*β*r2 downregulated chondrogenic ATDC5 cells couldn't be adjusted by BSHX formula treatment.

**Conclusion:**

By activation of TGF-*β* signaling, BSHX formula can promote articular cartilage repair by accelerating chondrocyte proliferation and maintaining chondrocyte phenotype, upregulate ECM accumulation, and inhibit matrix degradation.

## 1. Introduction

Articular cartilage is a highly specialized tissue; its unique feature owns just one cell type: the chondrocyte, which exhibits low proliferation activity and is imbedded in abundant extracellular matrix (ECM) [1]. Furthermore, this distinctive tissue plays an important role in joint movement, since it acts as a cushion in bone articulation which absorbs mechanical loading and also produces lubricating fluid to decrease joint friction. This built-for-life tissue exhibits poorly self-healing capacity in adulthood due to its nonvascular and noninnervated properties [2, 3]. However, repetitive mechanical loading and many pathologic factors can cause cartilage damage in the general and athletic populations [4, 5]. The cartilage lesion patients usually suffered from pain, swelling, and joint locking [6], which eventually lead to progressive damage and degenerative diseases, namely, osteoarthritis (OA) which is the leading cause of physical disability [7].

Numerous techniques are commonly used for cartilage repair. Microfracture acts as the most widely used marrow stimulation technique which could facilitate tissue regeneration and provide immediate relief of symptoms [8], but decreasing physical function after early improvement and increasing failure rate indicate the long-term clinical limitations of this operation [9]. Mosaicplasty such as autologous osteochondral transplantation and osteochondral allografting has reported some positive results but also presents disadvantages like procedure complexity, lack of integration, and instability [10–12]. Recently, tissue engineering has become an acceptable way for treating cartilage defect; although it may partly overcome these drawbacks, the optimal selection of seed cells and scaffold has not yet been well elucidated [13]. Moreover, inadequate biomechanical property often causes degradation of the implant. Generally speaking, none has so far been approved a promising way for cartilage regeneration.

Traditional Chinese medicine (TCM), which was applied for treating many disorders for thousands of years, has become a promising complementary and alternative medicine for osteoarthritis with limited side effects. It also shows strong effects in articular cartilage protection [14, 15]. According to the TCM theory, homeostasis maintenance of bone and cartilage and its regeneration rely on sufficient essence of kidney and liver [16]. Deficiency of kidney and liver combined with blood stasis can eventually cause OA-related cartilage damage. Bushenhuoxue (BSHX) formula, a tonifying kidney and promoting blood circulation decoction, has been clinically used for many years in OA treatment and yield positive outcome [17, 18]. Besides, we previously testified BSHX formula which inhibits cartilage degradation in OA mouse model [19], but it is not clear whether it can promote articular cartilage repair.

The transforming growth factor beta (TGF-*β*) signaling plays a vital role in cartilage homeostasis. Latent TGF-*β* binding to type II receptor (Tfg*β*r2) leads to recruitment and activation of type I receptor, which could phosphorylate its downstream target mothers against decapentaplegic homolog 2 (Smad2) and Smad3 finally regulating the expression of target genes such as collagen II (Col2), matrix metallopeptidase 13 (Mmp13), and Runt-related transcription factor 2 (Runx2). In mammalian cells, activation of TGF-*β* signaling regulates cell differentiation, promotes chondrocytes proliferation, and improves aggregation of Col2 and proteoglycan, preventing it from hypertrophy [20]. Therefore, pharmacological activation of TGF-*β* signaling has been proposed to accelerate articular cartilage repair [21, 22].

The purpose of the current study was the histological observation of the cartilage repair promoting effect of BSHX formula on rat cartilage defect model. We also testify this positive effect of BSHX formula by incubating chondrogenic ATDC5 cells in BSHX formula-mediated serum. For potential mechanism elucidation, we downregulate TGF-*β* signaling in chondrogenic ATDC5 cells by* Tgfβr2* siRNA to verify whether BSHX formula exerts articular cartilage repairing effect through this pathway.

## 2. Materials and Methods

### 2.1. Preparation of BSHX Formula

Ten herbal medicines in BSHX formula (Table 1) were provided by the First Affiliated Hospital of Zhejiang Chinese Medical University (Hangzhou, China). The exact chemical constituents and extraction process were described as follows. Briefly, eight abovementioned herb medicines were mixed in a ratio of 3:2:2:2:1:1:2:1 for aqueous extraction: Rehmannia glutinous (Liboscb), Eucommia ulmoides (Oliv.), Aconitum carmichaeli (Debx.), Lycium barbarum (L.), Cornus officinalis (Sieb.), Carthamus tinctorius (L.), Dioscoreae opposite (Thunb.), and Glycyrrhiza uralensis (Fisch.). Ethanol extraction was used for Cinnamomum cassia (Presl) and Prunus persica (Batsch.) in a ratio of 1:2. Finally, two types of extracts were mixed and concentrated to 2 g crude drug/ml for further use.

The analysis and quality control of BSHX were identified by the high-performance liquid chromatography assay (HPLC). Six of the major constituents including loganin, amygdalin, pinoresinol diglucoside, liquiritin, cinnamaldehyde, and hydroxysafflor yellow A were identified in our previous study [19].

### 2.2. Animals and Modeling

Twenty-four Sprague-Dawley rats (male, 12-week-old) were anesthetized by intraperitoneal injection of 1% pentobarbital sodium solution (0.3ml/100g). The knee joints were opened through a medial parapatellar incision, and femoral trochlea was exposed directly after patella dislocation. Electric drill was used for drilling a full thickness cartilage defect on the surface of trochlear groove (2mm diameter 2 mm depth), and incision was then sutured after patella dislocation was reduced. After surgery, all rats were treated with penicillin intramuscularly injected at 8U/rat for 3 consecutive days. All studies were approved by the Committee on the Ethics of Animal Experiments of Zhejiang Chinese Medical University.

### 2.3. Grouping and Drug Administration

All rats were divided into two groups randomly (n=12/group at 4w and 8w): model group and BSHX group. The day after surgery, BSHX formula was orally administered to BSHX group once a day at a dose of 0.7ml/100g body weight. The exact dose of oral administration was determined according to dosage conversion formula [23]. The same dose of 0.9% normal saline was fed to model group. All rats were sacrificed at 4w and 8w; the knee joint specimens were collected for further analysis.

### 2.4. Gross Conference and Histological Analysis

Knee joints were washed in 1× phosphate buffer saline (PBS) and photographs were then being captured in order to evaluate the status of cartilage by using the International Cartilage Repair Society (ICRS) cartilage repair assessment [24]. In this scoring system, lower score represents worse cartilage.

For histological analysis, specimens were fixed in 4% paraformaldehyde for 72 h, decalcified with 14% EDTA solution (PH=7.4) for about 30d, then dehydrated and embeded in paraffin. 3 *μ*m sagittal sections were stained with Alcian Blue Hematoxylin/Orange G (ABH) for gross structural analyzing. Mankin scoring system which graded cartilage on a scale of 0-13 was used to evaluate the cartilage lesion [25]. Higher score means worse repairing. Either ICRS assessment or Mankin scoring system was evaluated by two independent observers.

### 2.5. Immunohistochemistry (IHC) Analysis

After deparaffination and rehydration, 3 *μ*m sagittal sections were treated as follows: 0.3% hydrogen peroxide for 10 min to reduce endogenous peroxidase activity and 0.3% triton x-100 for increasing cell membrane permeability if necessary. Sections were then incubated in 0.1 mol/L citrate buffer (PH=4.0) in 60°C for 4 h antigen retrieval. 5% normal goat serum (Invitrogen, MD, USA) was used to block nonspecific staining for 20 min at room temperature. Sections were treated with anti-Col 2 (diluted 1:1000, Millipore, mab1330, USA), anti-Runx2 (diluted 1:200, abcam, ab76956, UK), anti-Mmp13 (diluted 1:200, abcam, ab39012, UK), and anti-pSMAD2 (diluted 1:100, abcam, ab188334, UK) primary antibody and incubated overnight at 4°C. Second antibody goat anti-mouse/rabbit antibody (diluted 1:1000) was added for 20 min, and diaminobenzidine solution (Invitrogen, MD, USA) was used for detecting positive staining while hematoxylin for counterstaining.

### 2.6. Preparation of BSHX Formula-Mediated Serum

Twenty Sprague-Dawley rats (8-week-old, weigh at 200±20 g) were randomly divided into BSHX group and control group. The rats in BSHX group underwent oral administration of BSHX (1 mg/10 g body weight/day) once a day for 7 days; meanwhile, the model group received 0.9% normal saline at the same dose. The blood samples were collected separately from the abdominal aorta; after being centrifuged at 3000 rpm/min at 4°C for 10 min, the serum of both groups was isolated and stocked at −80°C until required.

### 2.7. Cell Culture

ATDC5 cells (Riken Cell Bank, Ibaraki, Japan) underwent chondrogenesis by ITS (insulin, transferrin and selenous acid) (Gibco BRL, MD, USA) and were cultured in Dulbecco's modified Eagle's medium/Ham's F-12 medium (DMEM/F12; 1:1 mixture) which contained 10%Fetal bovine serum plus 50 U/ml penicillin and 50 mg/ml streptomycin for two weeks, incubated in 37°C, 5%CO_2_ humidified atmosphere.

### 2.8. Cell Proliferation Assay

Cell proliferation was tested by Cell Counting Kit-8 (CCK-8, Dojindo Laboratories, Kumamoto, Japan) following manufacturer's protocol. 100 *μ*l of chondrogenic ATDC5 cells suspensions (2 × 10^3^cells/well) were added into 96-well plates overnight. After the supernatant was removed, cells were then incubated in FBS-free medium which contains different concentrations of BSHX formula-mediated serum for 24 h. 10 *μ*l CCK8 solution was added to each well incubation for 3 h and the optimal concentration of BSHX formula-mediated serum was measured at 450 nm.

### 2.9. Transfection

Chondrogenic ATDC5 cells were transfected with Tgf*β*r2 small interfering RNA (Tgf*β*r2 siRNA, RiboBio, Guangzhou, China) by Lipofectamine RNAiMAX Transfection Reagent (Invitrogen, USA). The sequence of Tgf*β*r2 siRNA was 5′-CCUGUUGCCUGUGUGACUU-3′ (sense) and 3′-GGACAACGGACACACUGAA-5′ (antisense). The day before transfection, cells (1 × 10^5^cells/well) were plated into 6-well plates overnight and then incubated with transfection mixtures containing Tgf*β*r2 siRNA (final concentration 20 nM) or negative control siRNA (final concentration 20 nM) for 6 h. After transfection, the mixtures were replaced with complete culture medium. Transfection efficiency was measured at 24 h and 72 h by the expression level of Tgf*β*r2 mRNA through fluorescence quantitative PCR.

### 2.10. Quantitative Gene Expression Analysis

Total RNA was extracted from cells by using TRIzol (Invitrogen, CA, USA), isolated RNA was then reverse transcribed to cDNA using RevertAid First Strand cDNA Synthesis Kit (Invitrogen, CA, USA) according to manufacturer's procedure, fluorescence quantitative PCR was conducted by SYBR® Premix Ex Taq™ II (Takara, China), and the relative primers are present in (Table 2). The expression level of mRNAs was normalized to *β*-actin, respectively, as endogenous control and was calculated using the 2^-ΔΔCt^ method.

### 2.11. Western Blot Analysis

Cells were divided into four groups as follows: negative control siRNA (NC siRNA), NC siRNA + BSHX, Tgf*β*r2 siRNA, and Tgf*β*r2 siRNA + BSHX. All cells were lysed in lysis buffer to obtain total protein extracts, respectively. After being incubated on ice for 30 min, the extracts were separated with 12% SDS-PAGE gel and transferred to PVDF membranes. The membranes were then blocked in 5% milk for 1 h and incubated with different primary antibodies against phosphorylated-SMAD2/3 (diluted 1:1000, Cell Signaling Technology, #8828, USA) and SMAD2/3 (diluted 1:1000, Cell Signaling Technology, #8685, USA) overnight at 4°C. Next day, the membranes were incubated with goat anti-rabbit horseradish peroxidase-conjugated secondary antibody (diluted 1:5000; Abcam, ab6721, USA) at room temperature for 1 h. The densities of the bands were visualized by chemiluminescence.

### 2.12. Statistical Analysis

All data were described as mean ± standard deviation. Independent-samples t-test and one-way ANOVA test which were used in the present research were performed using SPSS software (version24.0, IBM Corporation, Armonk, NY, USA), and statistically significant changes were classified as significant (*∗*) while* p*<0.05 and highly significant (#) while* P*<0.01.

## 3. Results

### 3.1. BSHX Improves the Gross Appearance and Accelerates Tissue Regeneration of Cartilage Defects

**Figure 1:**
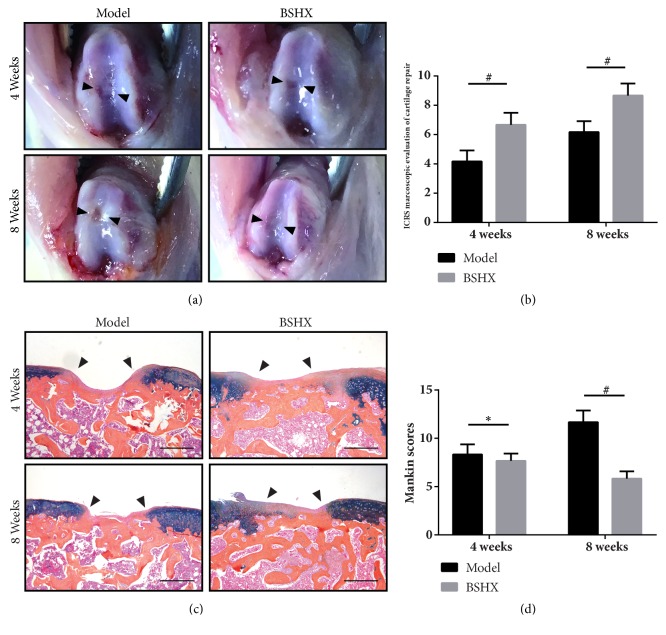
(a) Gross appearance of both model group and BSHX group at 4 and 8 weeks. (b) Assessment of ICRS cartilage repair score. (c) ABH staining for histological sections of both groups. Bar=50*μ*m. (d) Mankin score for cartilage repair. *∗P*<0.05, #*P*<0.01.

As shown in Figure 1(a), all specimens showed different degrees of cartilage damage. At 4 weeks, the defect area in the model group repaired slightly as it presented obvious lacuna with less regenerated tissue; meanwhile, plenty of regenerated tissue appeared in defect site of BSHX group but the surface was still rugged and lower than surrounding tissue. At 8 weeks, most parts of defect were filled by regenerated tissue covered with fibrillated surface. In model group, the surface of defects seemed rugged with big scratch on the junction between the defect sites and normal area. In BSHX group, the defected surface presented more smoothly and the regenerated tissue appeared similar to the native cartilage. The result of ICRS cartilage repair score was consistent with gross observation, and the macroscopic score of model group was higher than BSHX group in either 4w or 8w (*P*<0.01) (Figure 1(b)).

To figure out the cellular organization and ECM deposition of both groups, we then performed ABH staining (Figure 1(c)). In model group at 4w, the defects presented obvious lacuna which caved in new tissue containing thin fibrocartilage and plenty fibroblast morphologic cells. The defects were filled with new tissue over 50% depth at 8w; hardly any hyaline-like cartilage could be found in the regenerated surface. In BSHX group, the defects exhibited about 75% repair of the defect depth at 4w, the regenerated tissues were comprised of fibrocartilage and fibroblast morphologic cells. Astonishingly, the repaired tissue exhibited a slice of hyaline-like cartilage morphology and few glycosaminoglycans accumulations at 8w. BSHX group had lower Mankin score than model group in 4w and 8w (*P*<0.01) (Figure 1(d)). Altogether, BSHX group presents better cartilage morphology and higher surface regularity than model group.

### 3.2. BSHX Maintains the Homeostasis of Articular Cartilage and Chondrocytes

**Figure 2:**
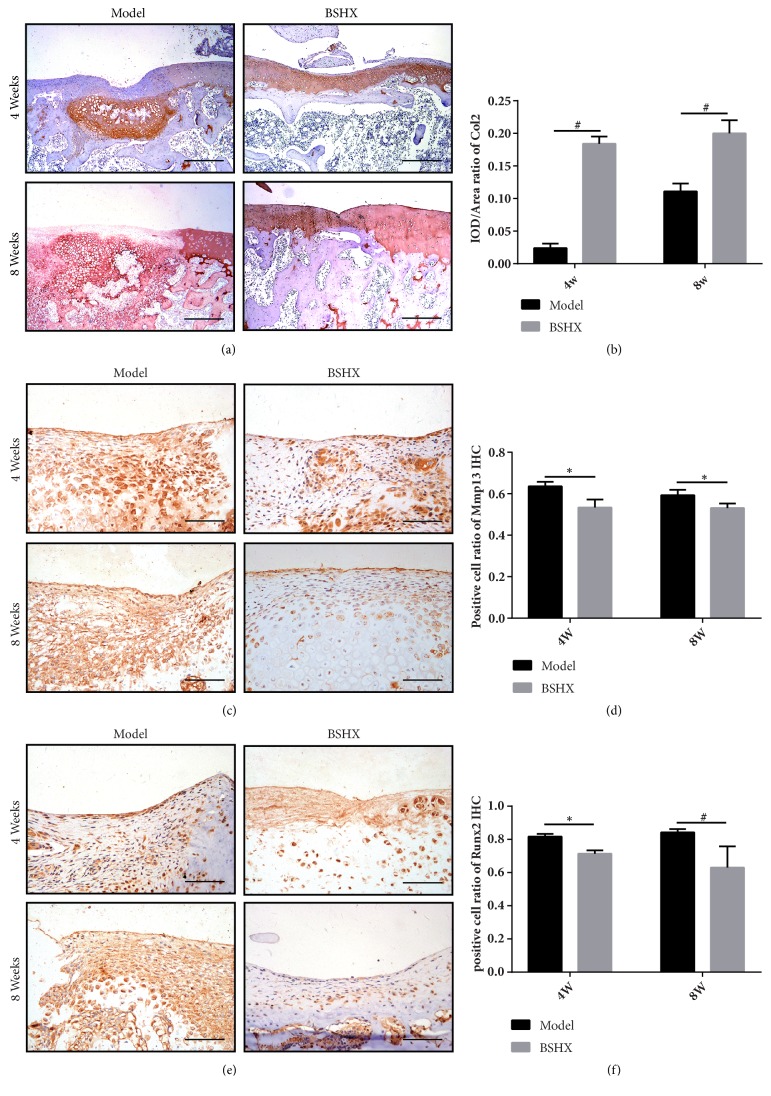
(a) Immunohistochemistry for Col II in both groups at 4 and 8 weeks, Bar=50*μ*m. (b) IOD/Area ratio of Col2 expression. (c) IHC for Mmp13, Bar=200*μ*m. (d) Positive cell ratio of Mmp13. (e) IHC for Runx2, Bar=200*μ*m. (f) Positive cell ratio of Runx2. *∗P*<0.05, #*P*<0.01.

To explain the changes identified by histology, we performed IHC on femur condylar of both groups. IHC for Col2 illustrated the expression of Col2 in newly formed tissue and surrounding normal cartilage was lower at both times in model group than BSHX group (*P*<0.01), and the regenerated tissue in BSHX group seemed almost similar to the surrounding cartilage (Figures 2(a) and 2(b)). We also performed IHC for MMP13, a key catabolic enzyme which took responsibility for cartilage damage and degradation. As expected, BSHX group showed lower expression in both 4w and 8w compared to model group (*P*<0.05) (Figures 2(c) and 2(d)). In addition, we detected a significant decrease in expression of Runx2 in BSHX group at 4w (*P*<0.05) and 8w (*P*<0.01) (Figures 2(e) and 2(f)), which marks chondrocytes hypertrophy.

**Figure 3:**
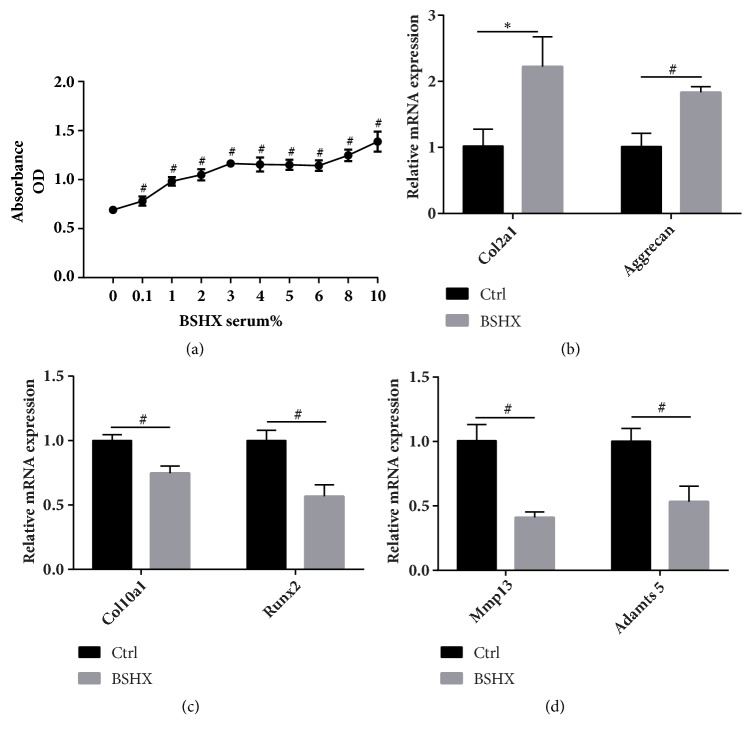
(a) Absorbance values of CCK8 test for cell proliferation and optimal BSHX-mediated serum concentration selection in chondrogenic ATDC5 cells after BSHX formula treatment. The expression alterations in Col2a1 and Aggrecan mRNA (b), Mmp13 and Adamts5 mRNA (c), Col10a1 and Runx2 mRNA (d). *∗P*<0.05, #*P*<0.01.

To extend our in vivo study, we then treated chondrogenic ATDC5 cells with BSHX formula containing culture medium. CCK-8 assay was performed for optimal serum concentration selection and cell proliferation assessment. Results showed obviously positive dosage-effect relation between cell proliferation and the concentration of serum; meanwhile, 10% BSHX-serum was the most efficient concentration (Figure 3(a)).

Fluorescence quantitative PCR was performed on RNA isolated from the cells of BSXH group and control group at 48 h. We first detected a significant elevation in the expression of Col2a1 and Aggrecan (Figure 3(b)), which presents ECM deposition. Next, we examined genes associated with cartilage degradation and saw a significant decrease in the level of Mmp13 and Adamts5 (Figure 3(c)). In addition, we looked at genes that define chondrocyte hypertrophy and terminal differentiation; we found that Col10a1 and Runx2 were all suppressed in BSHX group (Figure 3(d)). We believe BSHX formula can promote chondrocyte proliferation, maintain its characteristics, and prevent cartilage degeneration.

### 3.3. BSHX Exerts Its Articular Cartilage Repairing Effect through TGF-*β* Signaling Pathway

**Figure 4:**
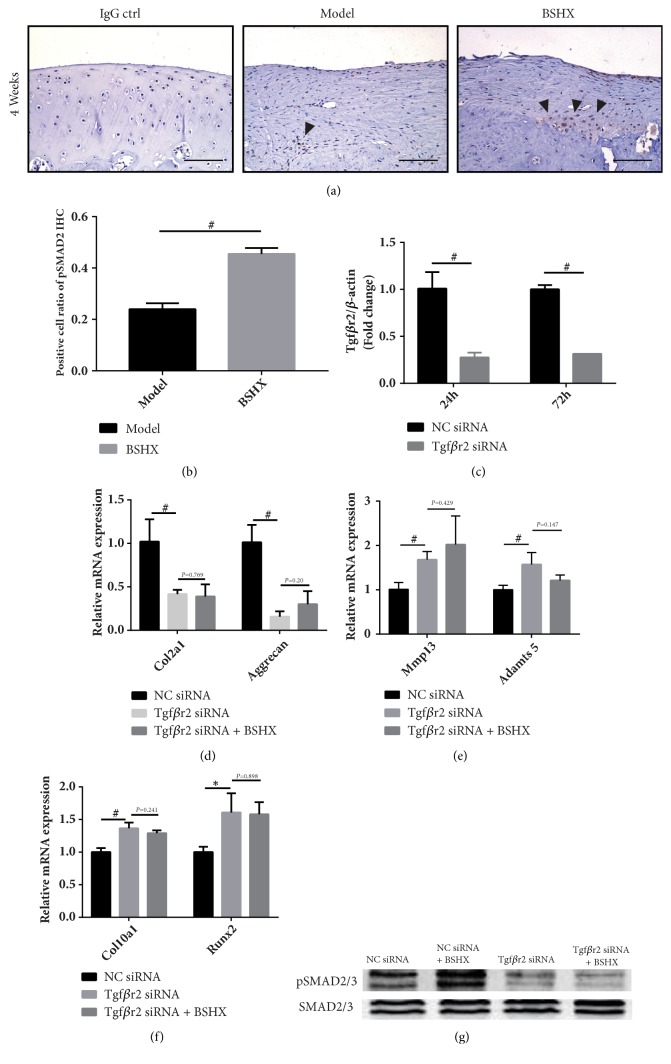
(a) IHC for pSMAD2, Bar=200*μ*m, black arrows point out the positive cells. (b) pSMAD2 positive cell ratio. Transfection efficiency of Tgf*β*r2 siRNA in Chondrogenic ATDC5 cells. (c) After transfected and BSHX formula treatment, ECM deposition (d), matrix degradation (e), and chondrocytes hypertrophy (f) related gene expression alterations in chondrogenic ATDC5 cells. (g) The protein expression of pSMAD2/3 in chondrogenic ATDC5 cells. *∗P*<0.05, #*P*<0.01.

Next, we wanted to determine if BSHX formula exerts the abovementioned cartilage repairing effect through TGF-*β* pathway. We first performed IHC for pSMAD2 in both groups at 4w; as expected, BSHX group presented more pSMAD2-positive cells than model group (P<0.01) (Figures 4(a) and 4(b)), which indicated the activation of TGF-*β* pathway.

To further explain this phenomenon, we then discussed it in vitro. The concentration of 20 nM Tgf*β*r2 siRNA was transfected into chondrogenic ATDC5 cells, and no-silence siRNA group as negative control. We assessed the expression of Tgf*β*r2 in these cells. We found the level of Tgf*β*r2 mRNA has been decreased about 70% of control values in 24 h and 72 h, whereas there was no significant difference between 24 h and 72 h in transfection efficiency (Figure 4(c)).

After transfection, the cells were divided into three groups: Tgf*β*r2 siRNA group and negative control siRNA group (NC siRNA) were treated with complete culture medium containing BSHX-free rat serum whereas Tgf*β*r2 siRNA plus BSHX group were treated with complete culture medium containing 10% BSXH formula-mediated serum; all groups were incubated at related culture medium for 48 h. As a result, compared to NC siRNA group, Tgf*β*r2 prominently decreased the expression of Col2a1 and Aggrecan, but BSHX formula could not reverse this tendency (Figure 4(d)). Similarly, the expression of Mmp13, Adamts5, Col10a1, and Runx2 was dramatically upregulated by partial blockage of TGF-*β* signaling while BSHX formula showed no effect in ECM protection and chondrocyte characteristic maintenance (Figures 4(e) and 4(f)). The protein expression of pSMAD2/3 was promoted by BSHX formula-mediated serum but downregulated by Tgf*β*r2 siRNA. However, after Tgf*β*r2 siRNA transfection, BSHX formula-mediated serum could not increase pSMAD2/3 expression (Figure 4(g)). The results insisted that BSHX formula exerts its cartilage regeneration promoting effect through TGF-*β* pathway.

## 4. Discussion

In the present study, experiments were performed to elucidate the impact of BSHX formula on articular cartilage repair in full-thickness cartilage defect rat model. Relative gross appearance and histological and immunohistochemistry examination revealed prominently articular cartilage repair promoting effect on cartilage lesion. We also found BSHX formula-mediated serum can accelerate chondrocyte proliferation and ECM synthesis meanwhile can prevent matrix degradation and chondrocyte hypertrophy on chondrogenic ATDC5 cells. However, the abovementioned biological role of BSHX formula was almost eliminated by downregulation of TGF-*β* signaling.

Repairing of articular cartilage lesion remains a challenging issue because of its avascular and aneural nature [26]. All reparative approaches were trying but failed to achieve the ultimate goal of regeneration and restoration of the hyaline cartilage in the defected area [27]. Cartilage-to-cartilage integration is extremely difficult to achieve because cartilage displays low metabolism and contains dense, antiadhesive ECM [28]. Without reasonable intervention, the newly formed tissue always presents fibrocartilage phenotype with inadequate biomechanical property compared to hyaline cartilage. As we saw in model group at 8w (Figure 1(a)), mismatches in this property result in strain disparities between neocartilage and surrounding tissue, leading to tissue degradation [10]. Therefore, it is urgent for clinicians and researchers to find a promising way to deal with this disorder.

Chinese medicine acting as a nonsurgical treatment is easy to perform and less invasive. Recently, these natural products have taken more attention in biomedical applications. Ample evidence suggests TCM as being effective in articular cartilage protection. For example, Zhang et al. described protective activity for cartilage via reducing the expression of Mmp13 and elevating Col2 with the TCM Yougui pills which were administered to mouse model induced by destabilization of the medial meniscus [29]. Wang et al. reported Bi-Qi capsule alleviated cartilage destruction by downregulating the expression of tumor necrosis factor-*α* (TNF-*α*) and Interleukin-18 (IL-18) [30]. TCM Qifangxibi granules exert chondroprotective effective through promoting CCAAT/enhancer binding protein-*α* methylation [31]. Moreover, our recent finding is consistent with these previous researches and extends the benefits of TCM to articular cartilage repair.

Full-thickness cartilage defect often implicates subchondral bone, and latent bone marrow mesenchymal stromal cells (BMSCs) and local growth factors may participate in the regeneration process through subchondral bone bleeding [32]. Due to BMSC pluripotency, subchondral bone regenerates and remodels up to a critical size through endochondral ossification which is consistent to our finding in model group at 4w (Figure 2(a)). BMSC can also be differentiated into chondrocyte, but unlike bone, articular cartilage regeneration relies on sufficient chondrocytes and cartilage-specific ECM deposition which could not be achieved by BMSC alone [33]. Chondrocytes occupy only 2% of the total tissue volume of the articular cartilage whereas ECM and water account for the rest [34]. Col2 and Aggrecan, two major solid components of ECM, accumulated at early stage of cartilage formation and remodeling due to its maturation [35]. The change in Col10 and Runx2 expression that was associated with chondrocytes terminal differentiation may alter the phenotypic state of chondrocytes [36, 37]; combined with increasing expression of several proteinases such as Mmp13 and Adamts5, it could degrade Col2 and Aggrecan [38, 39] and finally lead to degeneration of neocartilage tissue. Our results showed BSHX formula could provide material basis of cartilage repair by promoting chondrocytes proliferation and ECM assembly. Furthermore, it can also prevent the neocartilage from degradation by decreasing matrix degrading enzyme and chondrocytes hypertrophy factors.

In this study, significant articular cartilage repair promoting effect was found in cartilage defect rats treated with BSHX formula. Our data showed that BSHX formula could elevate protein expressional level of Col2 and pSMAD2 and decrease Mmp13 and Runx2 in cartilage defect rat. In treating chondrogenic ATDC5 cells, we also found that BSHX formula could promote cell proliferation and prevent it from hypertrophy and increase ECM synthesis meanwhile preventing it from degradation. To further elucidate the mechanism of BSHX formula articular cartilage repairing effects, we investigated the efficiency of BSHX formula in Tgf*β*r2 siRNA induced TGF-*β* signaling downregulated chondrogenic ATDC5 cells. As reported in previous researches, TGF-*β* signaling promotes chondrocytes to express ECM molecules, Col2, and Aggrecan to form cartilage tissue [40]. Deletion of Tgf*β*r2 resulted in upregulation of Runx2, Col10, Mmp13, and Adamts5 expression in chondrocytes [41]. In our study, the alteration of ECM deposition, chondrocyte hypertrophy-related genes, and pSMAD2/3 protein level observed in Tgf*β*r2 downregulated chondrogenic ATDC5 cells could not be adjusted by BSHX formula, indicating BSHX formula exerts its articular cartilage repairing effects through TGF-*β* signaling. However, Tgf*β*r2 siRNA could not completely impede the alteration of Adamts5 and Aggrecan expression induced by BSHX formula. One possible reason for this finding is that about 70% of Tgf*β*r2 gene was blocked in chondrogenic ADTC5 cells by Tgf*β*r2 siRNA; the transfection efficiency is close to the previous study [42]. 30% of Tgf*β*r2 remains normal in chondrocytes. Besides, considering the multitarget of TCM, BSHX formula might also regulate the expression of these genes through other signaling pathways which need further investigation.

## 5. Conclusion

In current study, we found that BSHX formula promotes articular cartilage repair as a natural TGF-*β* signaling activator via numerous ways, including acceleration of chondrocyte proliferation and maintaining chondrocyte phenotype, upregulates ECM accumulation, and inhibits matrix degradation either in or ex vivo. These findings enlighten us that TCM may be considered as an effective complementary and alternative medicine to cartilage damage; meanwhile, it encourages further research for this treatment.

## Figures and Tables

**Table 1:** The compositions of Bushenhuoxue (BSHX) formula.

Chinese name	Botanical name	Latin name	Parts used	Proportion
Shu Di Huang	Rehmannia glutinosa(Liboscb)	Rehmannia glutinous (Liboscb)	Root	17.4%
Du Zhong	Eucommia ulmoides (Oliv.)	Eucommiae cortex	Bark	11.8%
Fu Zi	Aconitum carmichaeli (Debx.)	Aconitilateralis radix preparata	Root	11.8%
Gou Qi	Lycium barbarum (L.)	Lyciifructus	Fruit	11.8%
Rou Gui	Cinnamomum cassia (Presl)	Cinnamomi cortex	Bark	5.9%
Shan Zhu Yu	Cornus officinalis (Sieb.)	Cornifructus	Fruit	5.9%
Tao Ren	Prunus persica (Batsch.)	Semen Persicae	Fruit	11.8%
Hong Hua	Carthamus tinctorius (L.)	Carthamus tinctorius (L.)	Corolla	5.9%
Shan Yao	Dioscoreae opposite (Thunb.)	Dioscoreae Rhizoma	Root	11.8%
Gan Cao	Glycyrrhiza uralensis (Fisch.)	Glycyrrhizae Radix et Rhizoma	Root	5.9%

**Table 2:** Primer name and sequences for PCR analysis.

Primer name	Sequence
*β*-actin forward	5′-GGAGATTACTGCCCTGGCTCCTA-3′
*β*-actin reverse	5′-GACTCATCTACTCCTGCTTGCTG-3′
Col2a1 forward	5′-TGGTCCTCT GGGCATCTCAGGC-3′
Col2a1 reverse	5′-GGTGAACCTGCTGTTGCCCTCA-3′
Mmp13 forward	5′-TTTGAGAACACGGGGAAGA-3′
Mmp13 reverse	5′-ACTTTGTTGCCAATTCCAGG-3′
Aggrecan forward	5′-CGCCACTTTCATGACCGAGA-3′
Aggrecan reverse	5′-TCATTCAGACCGATCCACTGGTAG-3′
Adamts5 forward	5′-CCAAATGCACTTCAGCCACGATCA-3′
Adamts5 reverse	5′-AATGTCAAGTTGCACTGCTGGGTG-3′
Col10a1 forward	5′-ACCCCAAGGACCTAAAGGAA-3′
Col10a1 reverse	5′-CCCCAGGATACCCTGTTTTT-3′
Runx2/Cbfa1 forward	5′-GAGGGCACAAGTTCTATCTGGA-3′
Runx2/Cbfa1 reverse	5′-GGTGGTCCGCGATGATCTC-3′

## Data Availability

The data used to support the findings of this study are available from the corresponding author upon request.
